# Development and validation of a fast ultra‐high‐performance liquid chromatography tandem mass spectrometry method for determining carbonic anhydrase inhibitors and their metabolites in urine and hair

**DOI:** 10.1002/dta.3055

**Published:** 2021-07-16

**Authors:** Alfredo Fabrizio Lo Faro, Anastasio Tini, Massimo Gottardi, Filippo Pirani, Ascanio Sirignano, Raffaele Giorgetti, Francesco Paolo Busardò

**Affiliations:** ^1^ Department of Excellence of Biomedical Sciences and Public Health University “Politecnica delle Marche” of Ancona Ancona Italy; ^2^ Comedical s.r.l. Trento Italy; ^3^ School of Law University of Camerino Camerino Italy

**Keywords:** carbonic anhydrase inhibitors, diuretics, hair, UHPLC–MS/MS, urine

## Abstract

A new, rapid, sensitive, and comprehensive ultra‐high‐performance liquid chromatography tandem mass spectrometry (UHPLC–MS/MS) method for quantifying diuretics (acetazolamide, brinzolamide, dorzolamide, and their metabolites) in human urine and hair was developed and fully validated. Twenty‐five milligrams of hair were incubated with 500‐μl M3® buffer reagent at 100°C for 1 h for complete digestion. After cooling, 1‐μl supernatant was injected onto chromatography system. Urine samples were simply diluted before injection. The chromatographic run time was short (8 min) through a column with a mobile phase gradient. The method was linear (determination coefficients always higher than 0.99) from limit of quantification (LOQ) to 500 ng/ml in urine and from LOQ to 10 ng/mg in hair. LOQs ranged from 0.07 to 1.16 ng/ml in urine and from 0.02 to 0.15 ng/mg in hair. No significant ion suppression due to matrix effect was observed, and process efficiency was always higher than 80%. Intra‐ and inter‐assay precision was lower than 15%. The suitability of the methods was tested with six urine and hair specimens from patients treated with acetazolamide, dorzolamide, or brinzolamide for ocular diseases or systemic hypertension. Average urine concentrations were 266.32 ng/ml for dorzolamide and 47.61 ng/ml for *N*‐deethyl‐dorzolamide (*n* = 3), 109.27 ng/ml for brinzolamide and 1.02 ng/ml for *O*‐desmethyl‐brinzolamide (*n* = 2), and finally, 12.63 ng/ml for acetazolamide. Average hair concentrations were 5.94 ng/mg for dorzolamide and 0.048 ng/mg for *N*‐deethyl‐dorzolamide (*n* = 3), 3.26 ng/mg for brinzolamide (*n* = 2), and 2.3 ng/mg for acetazolamide (*n* = 1). The developed method was simple and fast both in the extraction procedures making it eligible in high‐throughput analysis for clinical forensic and doping purposes.

## Graphical abstract

A new, rapid, sensitive, and comprehensive UHPLC–MS/MS method for determining acetazolamide, brinzolamide, dorzolamide, and their metabolites in human urine and hair was developed and fully validated.

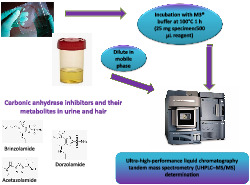

## 1 INTRODUCTION

Diuretics are pharmaceutical products commonly used to increase the renal function and stimulate the elimination of sodium. Therefore, they have long been used in the treatment of hypertension, edemas, electrolyte decompensation, and heart and renal failure.^1^, ^2^, ^3^ They are classified by their chemical structure, efficacy, speed of onset, or site of action. The most common classes are (i) loop diuretics, (ii) thiazides and related diuretics, (iii) potassium‐sparing diuretics, (iv) carbonic anhydrase inhibitors (CAIs), (v) osmotic diuretics, and (vi) miscellaneous diuretics.^4^ Among CAIs, the most commonly prescribed drugs are dorzolamide, brinzolamide, and acetazolamide, which are mainly used in ocular pathology for the treatment of glaucoma by reducing the intraocular pressure.^4^, ^5^, ^6^

CAIs inhibit the activity of carbonic anhydrase (enzymes that catalyze the interconversion between carbon dioxide and water and the dissociated ions of carbonic acid) in proximal convoluted tubules and prevent reabsorption of bicarbonates from renal tubule.^2^

For their pharmacological action, CAIs are included in class S5 (Diuretics and Masking Agents) of the prohibited list of substances of the World Anti‐Doping Agency (WADA), with the exception of drospirenone, pamabrom, CAIs in topical ophthalmic administration (e.g., dorzolamide and brinzolamide), and felypressin in local administration in dental anaesthesia,^7^ and therefore are banned in competitive sports mainly because they can be used to tamper with urine drug test results through dilution means. Hence, the identification of CAIs in urine and other biological matrices to provide orthogonal data on exposure (e.g., hair) can be of interest to document misuse.^8^

Presently, only few analytical methods^9^, ^10^, ^11^, ^12^, ^13^, ^14^, ^15^, ^16^, ^17^, ^18^ for the detection of CAIs in biological samples have been published in the international literature and none of them simultaneously determined the three main CAIs (dorzolamide, brinzolamide, and acetazolamide) and/or their metabolites.

Herein, a fast and simple method by ultra‐high‐performance liquid chromatography tandem mass spectrometry (UHPLC–MS/MS) was developed and fully validated to quantify dorzolamide, brinzolamide, acetazolamide, and the metabolites of the two first compounds, *N*‐acetyl‐dorzolamide, *N*‐deethyl‐dorzolamide, and *O*‐desmethyl‐brinzolamide, in human urine and hair. The method was then applied to samples collected from six authentic cases.

## 2 MATERIALS AND METHODS

### 2.1 Chemicals and reagents

Working standards of brinzolamide, dorzolamide, acetazolamide, *N*‐acetyl‐dorzolamide, *N*‐deethyl‐dorzolamide, *O*‐desmethyl‐brinzolamide, acetazolamide‐d_3_, and brinzolamide‐d_5_ were purchased from LGC (Queens Road, Teddington, Middlesex, UK) and stored at −20°C until use; acetazolamide‐d_3_ and brinzolamide‐d_5_ were used as internal standards (ISs). Methanol, dichloromethane, acetonitrile, formic acid, and ultra‐pure deionized water were obtained from Sigma‐Aldrich® (Milano, Italy). Ammonium formate buffer was prepared with ≥99% purity ammonium formate salt (Sigma‐Aldrich®) dissolved in water. M3®, an acidic buffer with a proprietary composition, was purchased from Comedical® s.r.l. (Trento, Italy). The solvents employed during extraction and in the chromatographic system were LC–MS grade.

### 2.2 Standard solution, calibration standards, and quality control samples preparation

Stock working solutions of brinzolamide, dorzolamide, acetazolamide, *N*‐acetyl‐dorzolamide, *N*‐deethyl‐dorzolamide, and *O*‐desmethyl‐brinzolamide at 10, 1, and 0.1 μg/ml were prepared in methanol. ISs working solutions with acetazolamide‐d_3_ and brinzolamide‐d_5_ at 1 μg/ml were prepared in methanol; final ISs concentration in spiked urine and hair was 5 ng/ml and 1 ng/mg, respectively. All solutions were stored at −20°C.

Calibration curve samples and low, medium, and high quality control (QC) samples were prepared with appropriate amounts of the stock working solutions in blank hair and urine samples, at the beginning of every analytical session. The concentrations of QCs were as follows: low QC = 31.5 ng/ml, medium QC = 87 ng/ml, high QC = 350 ng/ml for urine samples; low QC = 0.5 ng/mg, medium QC = 3.5 ng/mg, high QC = 7.5 ng/mg for hair samples.

### 2.3 Human samples

Blank human urine and hair samples were obtained from the laboratory storehouse of blank biological samples. In detail, drug‐free urine and hair were gently donated by university personnel involved in this study, signing an informed consent. All samples were prescreened by the below‐reported method to assess absence of the analytes under investigations.

Urine and hair specimens were collected from six patients treated with CAIs for different pathologies: three patients were treated with dorzolamide for ocular diseases, two patients were treated with brinzolamide, and one patient was treated with acetazolamide for systemic hypertension. These volunteers had been treated for a period ranging from 7 days to 20 years. All samples were collected at the Department of Excellent of Biomedical Science and Public Health (Università Politecnica delle Marche, Ancona, Italy). Urine samples were stored at −20°C, and hair samples (collected from occipital region) were stored at room temperature until analysis.

All the specimens were prescreened for the presence of any drug of abuse and pharmaceutical and analyzed to evaluate the robustness, accuracy, and suitability of the current method. The study was conducted in accordance with the ethical principles of the Declaration of Helsinki, and it received the ethics approval by the Institutional Ethical Committee (“CERM” no. 263/2020 Azienda Ospedaliero Universitaria Ospedali Riuniti Torrette di Ancona).

### 2.4 Sample treatment

Urine samples (100 μl) were fortified with 5‐μl ISs working solution in conical glass tubes and vortexed. After adding 5 ml of below‐described mobile phase A:B 95:5 (*v/v*), tubes were capped, vortexed for 15 s, and centrifuged at 15,000 g for 3 min. Supernatants (100 μl) were transferred into autosampler glass vials, prior to injection onto the chromatographic system.

Hair samples were washed twice with dichloromethane and dried under nitrogen at 45°C. An aliquot of 25 mg was finely cut into pieces (<5 mm) in glass tubes and fortified with 5‐μl ISs working solution. After adding 100‐μl M3® reagent, tubes were capped and incubated at 100°C for 1 h, for complete digestion. Then samples were cooled down at room temperature, and 1 ml was transferred into autosampler glass vial, prior to injection onto the chromatographic system.

### 2.5 UHPLC–MS/MS analysis

Samples analysis was performed with a Waters® Xevo® TQ‐S micro mass spectrometer (triple quadrupole) equipped with an electrospray ionization source operating in positive‐ion mode (ESI+) and interfaced with an ACQUITY UPLC® I‐Class (Waters®; Milano, Italy). Data were acquired with MassLynx® software Version 4.1 (Waters®).

Chromatography was carried out on an ACQUITY UPLC® BEH C_18_ column from Waters® (length: 50 mm, internal diameter: 2.1 mm, particle size: 1.7 μm). Run time was 8 min with a mobile phase gradient composed of 0.1% formic acid in 5‐mM ammonium acetate buffer (A) and 0.01% formic acid in methanol (B) at a flow rate of 0.35 ml/min.

Initial conditions were 5% B, held for the first 0.25 min, increased to 20% B from 0.26 to 3 min, increased to 95% from 3.1 to 5 min, held for 0.5 min, and returned 5% B to 8.00 min for re‐equilibration. Autosampler and oven temperatures were 10°C and 50°C, respectively. The injection volume was 3 μl for urine and 1 μl for hair samples.

The detection was performed with a triple quadrupole mass spectrometer operating in scheduled multiple reaction monitoring (MRM) mode, with two transitions for each analyte and IS, as reported in Table 1. MS parameters were optimized by infusing each standard individually in methanol; cone voltages, MRM transitions, and collision cell voltages were reported in Table 1. The source conditions were optimized as follows: capillary voltage, 0.5 kV; source temperature, 150°C; desolvation temperature, 650°C; cone flow rate, 20 L/h; and desolvation gas flow rate, 1200 L/h.

**TABLE 1:** Mass spectrometry parameters for analytes and internal standard under investigation

Analyte	Retention time (min)	MRM transitions					
		Quantifier MRM transition			Qualifier MRM transition		
		m/z	CV (v)	CE (eV)	m/z	CV (v)	CE (eV)
*N*‐Deethyl‐dorzolamide	0.90	297.1 > 135.1	30.0	26.0	297.1 > 199.0	30	14.0
Dorzolamide	1.00	325.1 > 135.1	25.0	28.0	325.1 > 199.0	25.0	18.0
Dorzolamide‐d_5_ (IS)	1.02	330.1 > 135.1	30.0	30.0	330.1 > 199.0	30.0	18.0
*O*‐Desmethyl‐brinzolamide	1.56	370.0 > 136.9	30.0	30.0	370.0 > 181.0	30.0	24.0
Acetazolamide‐d_3_ (IS)	2.60	226.1 > 73.3	30.0	34.0	226.1 > 165.0	30.0	22.0
Acetazolamide	2.60	223.1 > 73.3	30.0	34.0	226.1 > 163.0	30.0	22.0
Brinzolamide	4.12	384.0 > 217.1	30.0	22.0	384.0 > 281.0	30.0	16.0
*N*‐Acetyl‐dorzolamide	4.24	367.1 > 88.1	30.0	16.0	367.1 > 135.1	30.0	32.0

*Note*: Scan speed (dwell time and detection windows were adjusted accordingly).

Abbreviations: CE, collision energy; CV, cone voltage; IS, internal standard; MRM, multiple reaction monitoring.

### 2.6 Method validation

The method was fully validated in urine and hair according to most recent criteria for the validation of analytical toxicology.^19^, ^20^, ^21^, ^22^

Calibration points ranged from limit of quantification (LOQ) to 500 ng/ml in urine and from LOQ to 10 ng/mg in hair for each analyte. Linearity, sensitivity (limit of detection [LOD] and LOQ), precision, accuracy, and carryover were calculated injecting five different daily replicates of calibration point (five points for each calibration curve, including the LOQ) and five QC replicates (low QC = 31.5 ng/ml, medium QC = 87 ng/ml, high QC = 350 ng/ml in urine; low QC = 0.5 ng/mg, medium QC = 3.5 ng/mg, high QC = 7.5 ng/mg in hair) for three subsequent working days.

Least‐squares linear regression with the reversed square of the concentration of the analyte (1/*x* ^2^) was employed to define the calibration curve using the ratios of the peak area of analytes and IS.

The effect of three cycles of freezing at −20°C and thawing on five different aliquots of QC samples (urine and hair) were verified. The post‐extraction stability of the compounds under investigation was assessed under various conditions. The stability of five different extracted aliquots of QC samples (urine and hair) was assessed for 0, 1, 2, 4, 24, and 48 h in the light and dark at room temperature and at −20°C for up to 1 month.

The evaluation of ion suppression/enhancement due to matrix effect (ME), recovery (RE), and process efficiency (PE) was determined using the experimental design proposed by Matuszewski et al.^21^: Set 1 was composed of five replicates of analytes diluted in the mobile phase (low, medium, and high QC concentrations); Sets 2 and 3 were composed of five replicates of five different blank samples fortified with the analytes after and before extraction, respectively (low, medium, and high QC concentrations); for each analyte and concentration, ME was calculated by dividing mean peak areas of Set 2 by Set 1, RE was calculated by dividing mean peak areas of Set 3 by Set 2, and PE was calculated by dividing the mean peak areas of Set 3 by Set 1. Dilution integrity was verified for urine and hair over‐the‐curve samples with a concentration 10 and 50 times higher than the highest calibrators, with a dilution blank urine A:B 95:5 (*v/v*) before sample treatment. Calibration points and QC samples were prepared by two different staff members.

## 3 RESULTS

**FIGURE 1:**
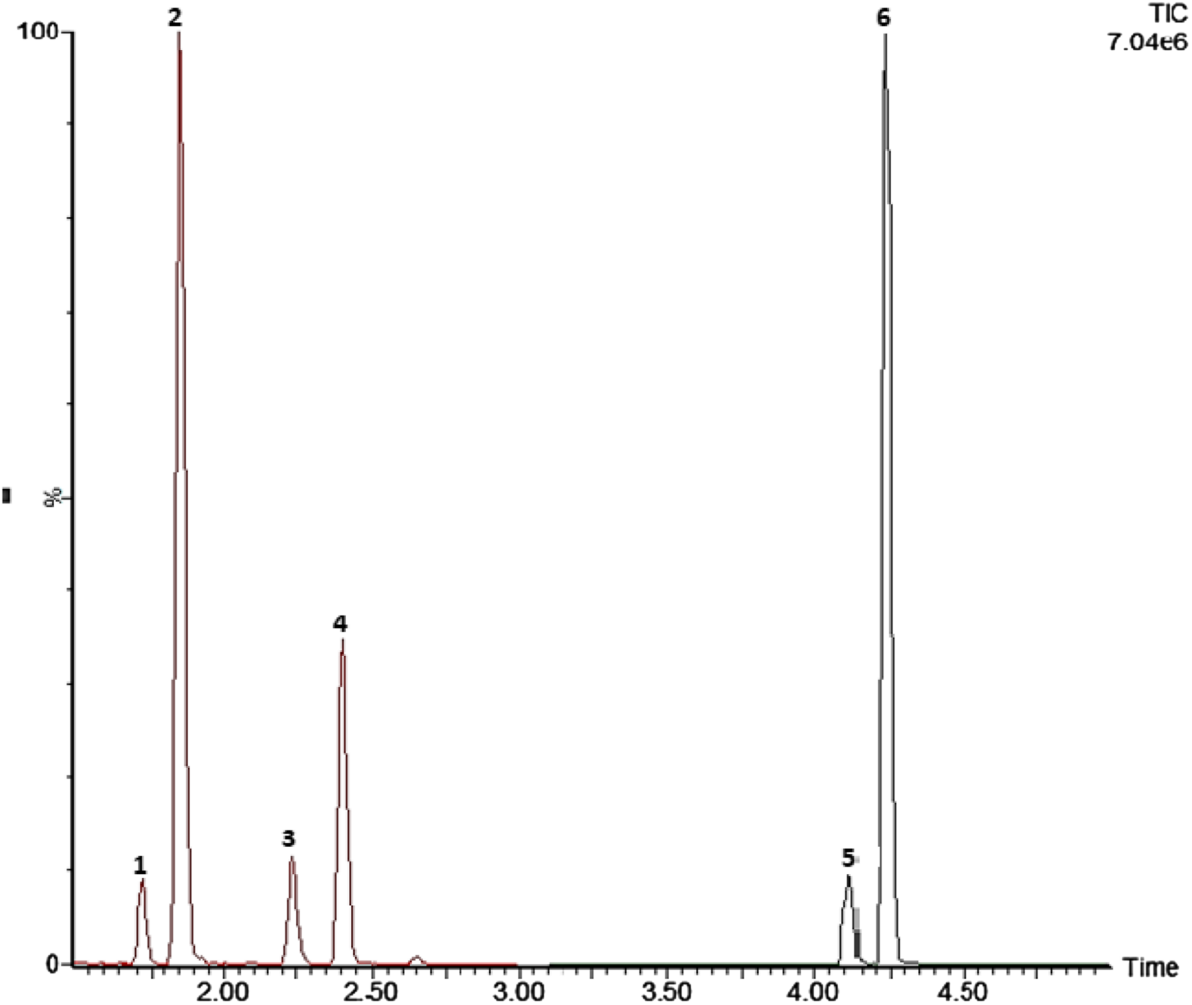
Ultra‐high‐performance liquid chromatography tandem mass spectrometry chromatogram of hair spiked with all the target analytes at a concentration of 0.02 ng/mg (limit of quantification). Deuterated standards peaks are not shown. 1, *N*‐deethyl‐dorzolamide; 2, dorzolamide; 3, acetazolamide; 4, *O*‐desmethyl‐brinzolamide; 5, brinzolamide; 6, *N*‐acetyl‐dorzolamide [Colour figure can be viewed at wileyonlinelibrary.com]

**FIGURE 2:**
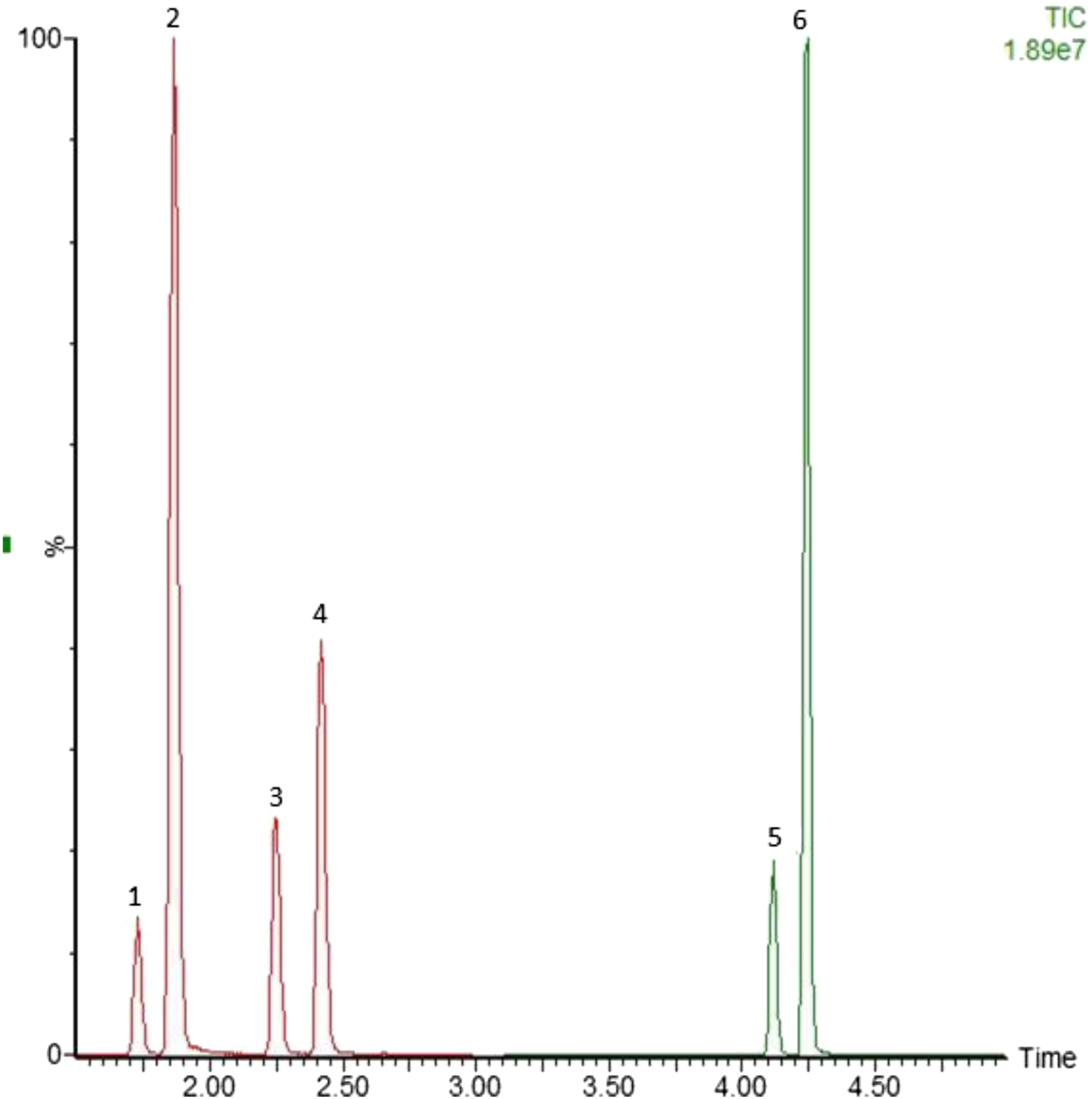
Ultra‐high‐performance liquid chromatography tandem mass spectrometry chromatogram of urine spiked with all the target analytes at a concentration of 0.07 ng/ml (limit of quantification). Deuterated standards peaks are not shown. 1, *N*‐deethyl‐dorzolamide; 2, dorzolamide; 3, acetazolamide; 4, *O*‐desmethyl‐brinzolamide; 5, brinzolamide; 6, *N*‐acetyl‐dorzolamide [Colour figure can be viewed at wileyonlinelibrary.com]

Representative extracted ion chromatograms obtained after extraction of blank urine and hair samples fortified with the analytes at 0.07 ng/ml and 0.02 ng/mg (LOQ), respectively, are shown in Figures 1 and 2. Validation parameters in human urine and hair are reported in Tables 2 and 3. The analysis of six batches of blank samples showed no interfering peak in the MRM traces for dorzolamide, brinzolamide, acetazolamide, their metabolites, and ISs in human samples. Blank responses could not be distinguished from the detector noise (signal‐to‐noise ratio < 3) for all analytes and ISs and were all below 15% of the LLOQ.

**TABLE 2:** Validation parameters for the analytes under investigation in urine

Compound	Mean regression curve and intercept	LOD (ng/ml)	LOQ (ng/ml)	Accuracy (% error)			Intra‐assay precision (% CV)			Inter‐assay precision (% CV)			Recovery (%) *n* = 15	Matrix effect (%) *n* = 15	Process efficiency (%) *n* = 15
				Low QC *n* = 15	Mid QC *n* = 15	High QC *n* = 15	Low QC *n* = 15	Mid QC *n* = 15	High QC *n* = 15	Low QC *n* = 15	Mid QC *n* = 15	High QC *n* = 15			
*N*‐Deethyl‐dorzolamide	*y* = 0.0085*x* + 0.0071	0.07	0.24	8.6	6.6	4.0	3.0	1.8	0.8	7.7	4.9	3.5	81.0	92.9	80.0
Dorzolamide	*y* = 0.0079*x* + 0.0237	0.11	0.38	3.8	4.1	1.3	2.3	2.1	1.0	1.6	2.2	1.9	96.5	78.8	79.9
Acetazolamide	*y* = 0.008*x* + 0.0307	.13	0.43	5.7	3.0	4.1	2.8	3.0	2.2	4.9	3.3	2.2	91.2	87.4	73.3
*O*‐Desmethyl‐brinzolamide	*y* = 0.0081*x* + 0.0193	0.35	1.16	3.4	3.3	2.1	1.3	1.3	0.9	3.2	2.0	0.9	97.2	88.2	82.7
Brinzolamide	*y* = 0.0083*x* + 0.028	0.02	0.07	10.6	1.9	4.3	0.6	2.4	3.7	7.4	1.6	3.3	98.1	97.0	95.1
*N*‐Acetyl‐dorzolamide	*y* = 0.0092*x* + 0.005	0.17	0.55	6.9	4.4	14.6	1.9	4.3	1.1	1.8	5.3	9.3	86.0	96.5	82.5

*Note*: Low, medium, and high quality control (QC) samples contained all standards at 31.5, 87, and 350 ng/ml, respectively. Analytical recovery, matrix effect, and process efficiency are displayed as mean value of low, medium, and high QC values.

Abbreviations: CV, coefficient of variation; LOD, limit of detection; LOQ, limit of quantification.

**TABLE 3:** Validation parameters for the analytes under investigation in hair

Compound	Mean regression curve and intercept	LOD (ng/mg)	LOQ (ng/mg)	Accuracy (% error)			Intra‐assay precision (% CV)			Inter‐assay precision (% CV)			Recovery (%) *n* = 15	Matrix effect (%) *n* = 15	Process efficiency (%) *n* = 15
				Low QC *n* = 15	Mid QC *n* = 15	High QC *n* = 15	Low QC *n* = 15	Mid QC *n* = 15	High QC *n* = 15	Low QC *n* = 15	Mid QC *n* = 15	High QC *n* = 15			
*N*‐Deethyl‐dorzolamide	*y* = 0.0067*x* + 0.008	0.01	0.04	14.6	5.4	5.0	2.1	5.0	1.6	14.6	5.2	6.7	96.5	78.0	75.0
Dorzolamide	*y* = 0.0081*x* + 0.0511	0.01	0.02	9.5	4.6	1.6	0.8	4.5	0.9	12.4	4.7	2.1	97.0	92.7	90.1
Acetazolamide	*y* = 0.0075*x* + 0.0117	0.01	0.03	4.6	6.4	1.4	4.6	1.2	0.9	7.2	1.5	0.9	97.9	90.5	88.7
*O*‐Desmethyl‐brinzolamide	*y* = 0.0069*x* + 0.0433	0.05	0.15	4.4	9.1	1.7	5.1	6.1	0.8	6.2	5.6	1.5	97.4	91.2	89.0
Brinzolamide	*y* = 0.008*x* + 0.0322	0.02	0.06	2.1	6.5	3.5	1.6	3.0	1.7	1.9	5.0	4.2	99.0	96.6	95.6
*N*‐Acetyl‐dorzolamide	*y* = 0.0098*x* + 0.007	0.01	0.02	4.1	2.6	1.4	5.8	4.1	1.4	4.8	3.1	1.3	99.0	89.9	90.0

*Note*: Low, medium, and high quality control (QC) samples contained all standards at 0.5, 3.5, and 7.5 ng/mg, respectively. Analytical recovery, matrix effect, and process efficiency are displayed as mean value of low, medium, and high QC values.

Abbreviations: CV, coefficient of variation; LOD, limit of detection; LOQ, limit of quantification.

Carryover was evaluated by the analysis of blank matrix samples (urine and hair) after the highest point of calibration curve, and no analytes carryover was observed.

Separation of the analytes and ISs was completed in a total run time of 8 min, including LC column flushing and equilibration.

The method exhibited good linearity and showed coefficients of determination (*r* ^2^) always better than 0.99. The percentage of relative error for all the calibration points of the curve was less than 10%.

LOD ranged from 0.02 to 0.17 ng/ml in urine and from 0.01 to 0.05 ng/mg in hair, and LOQ ranged from 0.07 to 1.16 ng/ml in urine and from 0.02 to 0.15 ng/mg in hair; accuracy and precision were within ±20% of the target at the LOQ. REs ranged from 81% to 98.1% (relative standard deviation [RSD] between 1.5% and 5.6%) in urine and from 96.5% to 99% (RSD between 4% and 12%) in hair. PE was always better than 73% and ion suppression due to ME within 12% (RSD between 3% and 7.6% in urine and between 6.5% and 10% in hair).

The effect of freezing at −20°C and thawing cycles on QC samples did not significantly alter the compounds concentration in biological matrices (differences from controls less than 10%). Furthermore, the post‐extraction stability of the substances under investigation was proved at room temperature and at −20°C, with differences in concentrations less than 10% control samples.

### 3.1 Real samples

**FIGURE 3:**
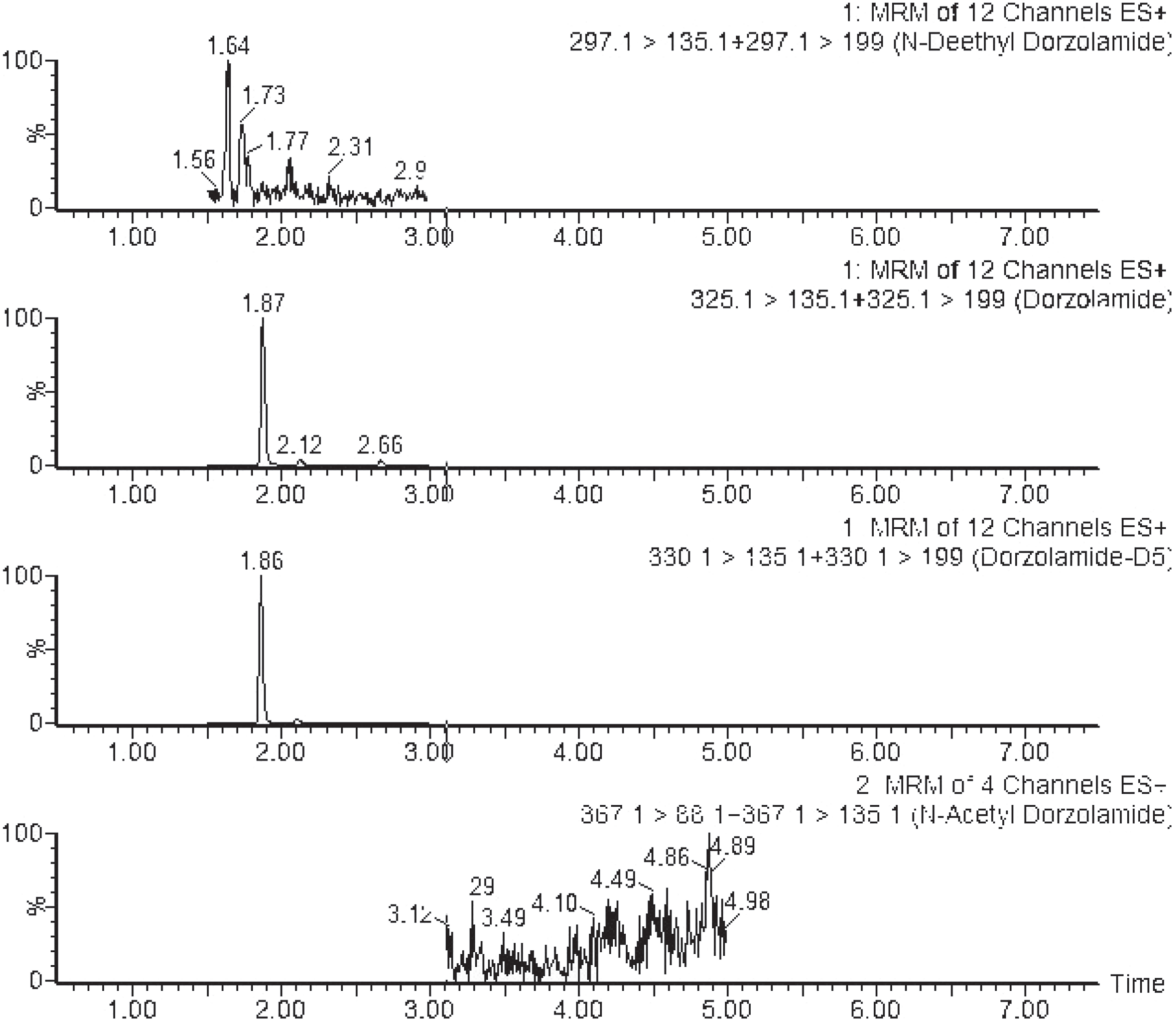
Ultra‐high‐performance liquid chromatography tandem mass spectrometry chromatogram of hair sample collected from a volunteer and containing 0.09 ng/mg of *N*‐deethyl‐dorzolamide (retention time 1.64 min) and 16.21 ng/mg of dorzolamide (retention time 1.87 min). *N*‐Acetyl‐dorzolamide was not detected. MRM, multiple reaction monitoring

**FIGURE 4:**
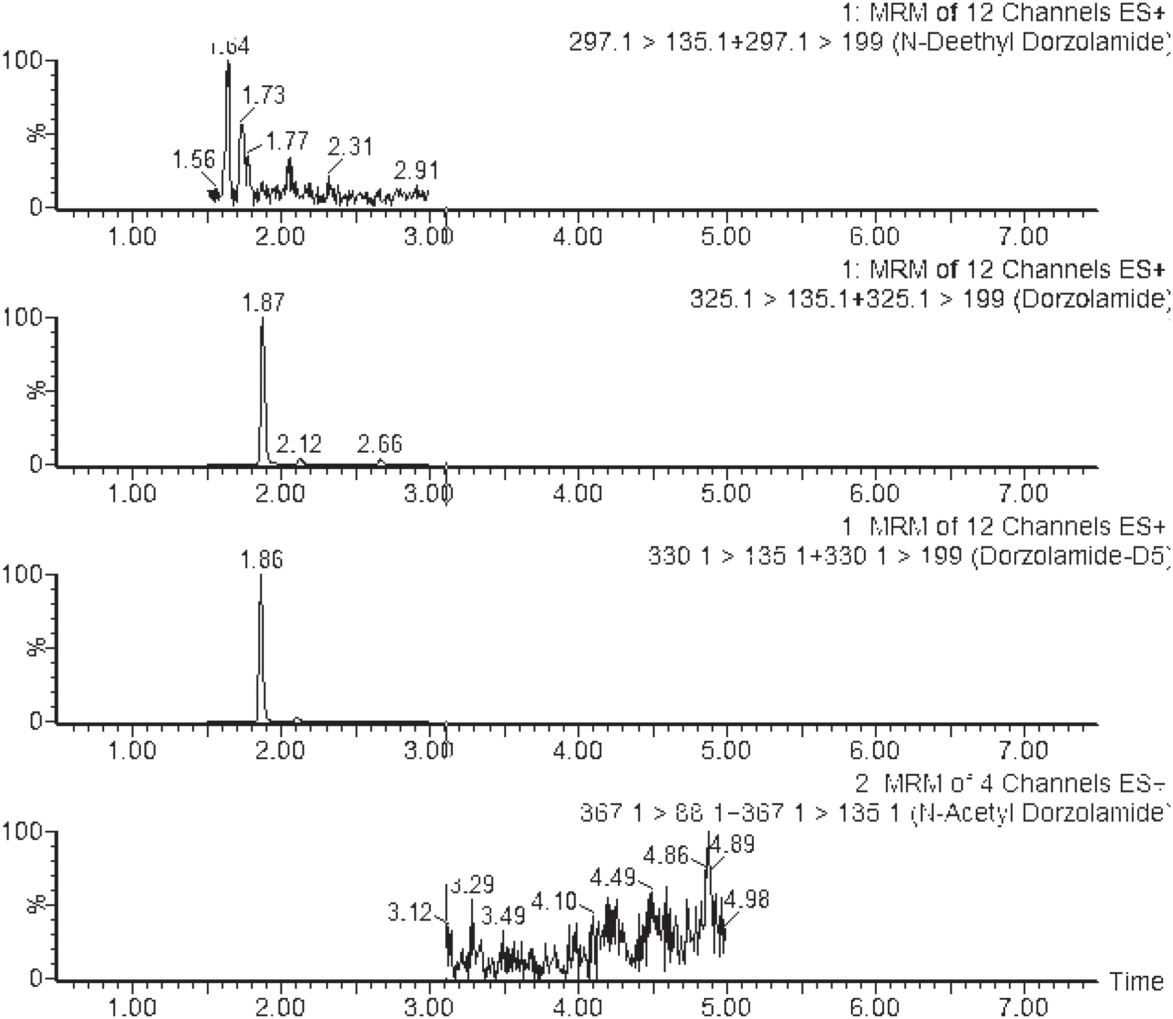
Ultra‐high‐performance liquid chromatography tandem mass spectrometry chromatogram of urine sample collected from a volunteer and containing 4.63 ng/ml of *N*‐deethyl‐dorzolamide (retention time 1.64 min) and 20.08 ng/ml of dorzolamide (retention time 1.87 min). *N*‐Acetyl‐dorzolamide was not detected. MRM, multiple reaction monitoring

Target analyte concentrations in authentic samples are reported in Table 4. Average (standard deviation [SD]) concentrations in urine samples were 266.32 ng/ml (249.35) for dorzolamide, 47.61 ng/ml (23.15) for *N*‐deethyl‐dorzolamide (*n* = 3), 109.27 ng/ml (0.036) for brinzolamide, 1.02 ng/ml (0.41) for *O*‐desmethyl‐brinzolamide (*n* = 2), and finally, 12.63 ng/ml for acetazolamide (*n* = 1). Average hair concentrations were 5.94 ng/mg (8.91) for dorzolamide, 0.14 ng/mg (0.13) for *N*‐deethyl‐dorzolamide (*n* = 3), 3.26 ng/mg (4.2) for brinzolamide (*n* = 2), and finally, 2.3 ng/mg for acetazolamide (*n* = 1). *O*‐Desmethyl‐brinzolamide and *N*‐acetyl‐dorzolamide were not detected in hair. Representative extracted ion chromatograms of real urine and hair samples are show in Figures 3 and 4.

**TABLE 4:** Concentration of diuretics and their metabolites in authentic specimens

Sample ID	Matrix	*N*‐Deethyl‐dorzolamide	Dorzolamide	Acetazolamide	*O*‐Desmethyl‐brinzolamide	Brinzolamide	*N*‐Acetyl‐dorzolamide
#1	U	4.63	20.08	‐	‐	‐	‐
	H	0.09	16.21	‐	‐	‐	‐
#2	U	100.85	518.68	‐	‐	‐	<LOQ
	H	0.025	0.23	‐	‐	‐	‐
#3	U	37.37	260.20	‐	‐	‐	0.08
	H	0.03	1.36	‐	‐	‐	‐
#4	U	‐	‐	‐	0.73	36.11	‐
	H	‐	‐	‐	‐	6.24	‐
#5	U	‐	‐	‐	1.31	182.43	‐
	H	‐	‐	‐	‐	0.29	‐
#6	U	‐	‐	12.63	‐	‐	‐
	H	‐	‐	2.3	‐	‐	‐

*Note*: Concentration in urine is indicated in ng/ml; concentration in hair is indicated in ng/mg.

Abbreviations: H, hair; LOQ, limit of quantification; U, urine.

In the time of our study, there was no information from international literature regarding acetazolamide metabolism in human model, nor was any drug metabolite commercially available. For this reason, we only analyzed parent compound in biological matrices. Same in vitro experiments on acetazolamide's metabolism are ongoing to better clarify this study limitation.

## 4 DISCUSSION AND CONCLUSION

To date, few analytical methods, based on liquid and gas chromatography, were published in the international literature to quantify CAIs in biological and nonbiological matrices such as blood, plasma, vitreous humor, capsules, and powder.^10^, ^11^, ^12^

We developed a method to detect parent drugs and metabolites in urine samples because this biological matrix is the only one allowed for doping control. To date, no cutoff values have been established to disclose the use of CAIs for doping purposes, and our study represents a preliminary step to solve this gap. Nevertheless, we also set up a method for hair samples because in case of judicial debate on a doping offense, this last matrix is used to exclude chronic use of CAIs and demonstrate a causal unconscious intake.

With respect to urine and hair samples, only a few methods published concerning urine samples and none for hair specimens. In 2020, Begou et al. developed a gas chromatography‐negative‐ion chemical ionization‐mass spectrometry method to quantify acetazolamide and other sulfonamides in human urine. The method consisted in a 9‐min analysis with sample preparation based on evaporation to dryness of urine aliquots and its subsequent derivatization. The LODs were 222.24 ng/ml; intra‐ and inter‐assay precision and accuracy were 0.3%–4.2% and 95.3%–109%, respectively.^14^ In 2006, Morra et al. validated a gas chromatography/electrospray ionization‐mass spectrometry method for quantifying acetazolamide along with 15 other diuretics and masking agents in human urine. Sample preparation involved a liquid–liquid extraction and a derivatization with methyl iodide for 2 h, and the chromatographic run is completed in less than 4 min, LODs ranging from 0.6 to 2800 ng/ml.^15^ In 2005, Beyer et al. developed a screening method for detecting acetazolamide and other diuretics in human urine after extractive methylation and GC/EI‐MS analysis with a 8‐min separation. LOD ranged from 0.001 to 5 mg/L.^16^ Finally, back in 1991, Lisi et al. analyzed bumetanide, ethacrynic acid, hydrochlorothiazide, chlorothiazide, and acetazolamide in human urine by GC/MS–MS and derivatization by extractive methylation directly from the urine. LODs ranged from 0.03 to 0.10 μg/ml.^17^ All the methods described are characterized by a lengthy pretreatment of the samples making these methods long lasting to be included as a routine analysis in toxicological laboratories.

Differently from the above‐reported methods, we developed and validated an assay for simultaneously quantifying dorzolamide, brinzolamide, acetazolamide, and their metabolites, *N*‐deethyl‐dorzolamide, *N*‐acetyl‐dorzolamide, and *O*‐desmethyl‐brinzolamide, in human urine and hair. This method is simple and fast both in the extraction procedures (dilute and shoot for urine samples and 1‐h digestion with M3® reagent for hair sample) making it eligible in high‐throughput analysis for clinical forensic and doping purposes. A complete washing of the instrument and the injector with the mobile phase at the end of a working day were performed.
